# Exploring ADHD Symptoms and Associated Impairment Across Development

**DOI:** 10.1177/10870547211025612

**Published:** 2021-06-24

**Authors:** Ayako Niina, Olga Eyre, Robyn Wootton, Evie Stergiakouli, Anita Thapar, Lucy Riglin

**Affiliations:** 1 Division of Psychological Medicine and Clinical Neurosciences, MRC Centre for Neuropsychiatric Genetics and Genomics, Cardiff University, UK; 2 MRC Integrative Epidemiology Unit, University of Bristol, Bristol, UK; 3 Nic Waals Institute, Lovisenberg Diaconal Hospital, Oslo, Norway; 4 Population Health Sciences, Bristol Medical School, University of Bristol, Bristol, UK

**Keywords:** ADHD, adult, ALSPAC, impairment, longitudinal, prospective

## Abstract

**Objective:**

ADHD symptoms typically decline with age, but less is known about whether the presentation of specific ADHD symptoms differs across development. This study aimed to examine the frequency and associated impairment of specific ADHD symptoms in childhood, adolescence and young adulthood.

**Method:**

A prospective, longitudinal cohort, the Avon Longitudinal Study of Parents and Children, was utilised (N=2327). ADHD symptoms and impairment were assessed using the Development and Well Being Assessment at ages 7, 15 and 25.

**Results:**

Specific ADHD symptom frequencies and their associated impairment varied across development for the majority of symptoms, although *easily distracted* was one of the most commonly reported symptoms at each age, and *difficulty sustaining attention* was consistently associated with high levels of impairment.

**Conclusion:**

These findings suggest differences in the presentations of ADHD symptoms across development: current understanding of how ADHD presents in childhood/adolescence may not be generalisable to young adulthood.

## Exploring ADHD Symptoms and Associated Impairment Across Development

Attention Deficit Hyperactivity Disorder (ADHD) is a neurodevelopmental disorder characterised by hyperactive-impulsive and inattentive symptoms that are inconsistent with the individual’s development and lead to functional impairment (American Psychiatric Association, 2013; National Institute of Health and Clinical Excellence, 2018; Thapar & Cooper, 2016). When defined categorically, ADHD is estimated to have a prevalence of 3.4% in children and adolescents (Polanczyk et al., 2015). However, ADHD symptoms behave as continuous traits - for example subthreshold symptoms show associations with the same risk-factors and adverse outcomes as ADHD diagnosis (Polanczyk et al., 2015; Thapar & Cooper, 2016). An estimated 18.5% of children and adolescents who show possible functional impairments resulting from ADHD symptoms do not meet full ADHD diagnostic criteria (Kirova et al., 2019; Thapar & Cooper, 2016).

Current diagnostic criteria for ADHD in adulthood require evidence of ADHD symptoms to be present since childhood. An estimated 65% of those diagnosed in childhood show persistence of ADHD-related difficulties when followed-up into young adulthood, and 15% still meet full diagnostic criteria (Faraone et al., 2006). DSM-5 diagnostic criteria acknowledge some developmental differences in ADHD in that fewer symptoms are required for diagnosis in adulthood compared to childhood (five and six respectively) (American Psychiatric Association, 2013). Research also suggests developmental differences in the presentation of ADHD symptoms. While there is a general trend for ADHD symptoms to decline with age (Faraone et al., 2006; Thapar & Cooper, 2016), evidence from clinical and non-clinical cohorts suggest that hyperactive-impulsive symptoms are more common in childhood and begin to decline in adolescence. In adolescence, inattentive symptoms are more common, but then decline into adulthood. This results in similar levels of hyperactive-impulsive and inattentive symptoms by adulthood (Lahey & Willcutt, 2010; Larsson et al., 2011).

To date longitudinal studies investigating ADHD symptom changes over time have predominantly examined the prevalence of hyperactive-impulsive compared to inattentive symptoms across development. A better understanding of the presentation of ADHD across development would be aided by investigation of specific ADHD symptoms: indeed, the studies that have examined specific ADHD symptoms suggest variation in presentation with age. For example, a population twin study of children and adolescents that examined ADHD symptom stability over a five-year period found that inattentive symptoms tended to be more stable than hyperactive-impulsive ones. However, this was not uniform, with some inattentive symptoms showing relatively high levels of decline over this time (e.g. *not seeming to listen*) (Todd et al., 2008). The wide age range of this study (7-19 years at baseline) limited the extent to which longitudinal comparisons could be made across specific developmental periods.

Another community sample (Holbrook et al., 2016) with a narrower age range following children (age 5-13 years) over a six-year period, across five assessments, found the prevalence of all nine inattention symptoms to be fairly stable across development, although some became less common, for example with being *easily distracted* was one of the most common symptoms in childhood but not adolescence. They also showed a fairly uniform attenuation of the nine hyperactive-impulsive symptoms, although the prevalence of *run about or climb excessively* - one of the most common symptoms in early childhood (age 5-8 years) was less common even by middle childhood (age 9-11 years). Thus, in addition to evidence of a general decline in symptoms, there is some evidence that the predominance of specific ADHD symptoms may change across development. However, longitudinal research is needed to compare specific symptom prevalence across childhood, adolescence and into young adulthood.

In addition to the prevalence of specific ADHD symptoms across development, it also is not yet clear whether the impairment associated with different ADHD symptoms differs across different developmental periods. One study investigating associations between ADHD symptoms and impairment across three school-based population samples covering early childhood (age 4-6 years), middle childhood (age 8-12 years) and adolescence (approximately 14-18 years) (Zoromski et al., 2015) observed differences according to age whereby hyperactive-impulsive symptoms showed greater association with impairment in early childhood but inattentive symptoms were associated with greater impairment in middle childhood and adolescence. They also found that while some symptoms were associated with impairment across age groups (e.g. *does not seem to listen* and *avoids tasks*), there was some variation with others predicting greater impairment at specific ages (e.g. *being on the go* in early childhood, *leaves seat* in middle childhood, and *does not follow through* in adolescence). Thus, both the frequency and associated impairment of specific ADHD symptoms may vary across development. However, longitudinal research examining the same individuals into young adulthood is lacking.

Our aims were to utilise prospective longitudinal data from a population cohort to (1) investigate the presentation of specific ADHD symptoms in childhood, adolescence and young adulthood (ages 7, 15 and 25 years) using the same measure and informant at each age, and (2) examine the level of impairment associated with specific symptoms at these time points.

## Method

### Sample

We analysed data from the Avon Longitudinal Study of Parents and Children (ALSPAC). In this study, pregnant women resident in Avon, UK with expected dates of delivery 1st April 1991 to 31st December 1992 were invited to take part. The initial number of pregnancies enrolled was 14,541 (for these at least one questionnaire has been returned or a “Children in Focus” clinic had been attended by 19/07/99). Of these initial pregnancies, there was a total of 14,676 foetuses, resulting in 14,062 live births and 13,988 children who were alive at 1 year of age (Boyd et al., 2013). Part of this data was collected using REDCap (Harris et al., 2019; Harris et al., 2009). Ethical approval for the study was obtained from the ALSPAC Law and Ethics Committee and Local Research Ethics Committees. Informed consent for the use of data collected via questionnaires and clinics was obtained from participants following the recommendations of the ALSPAC Ethics and Law Committee at the time. Please note that the study website contains details of all the data that is available through a fully searchable data dictionary and variable search tool: http://www.bristol.ac.uk/alspac/researchers/our-data/. Further details of the study, measures and sample can be found elsewhere (Boyd et al., 2013; Fraser et al., 2013; Northstone et al., 2019). Where families included multiple births, we included the oldest sibling.

### Measures

ADHD symptoms and impairment were assessed using the parent-rated Development and Well-Being Assessment (DAWBA) (Goodman et al., 2011) at ages 7 (childhood), 15 (adolescence) and 25 years (young adulthood) (Goodman et al., 2000). The DAWBA is a structured diagnostic interview based on DSM-IV and ICD-10 diagnostic criteria and has been used extensively in epidemiological studies. While self-reports would be the primary data used to assess ADHD symptoms in adult mental health services, parent-reports are typically used in childhood and have also been found to be important informants into early adulthood (Barkley et al., 2002). To enable the use of the same measure and informant across ages we therefore used parent-reports at all three ages.

The 18 ADHD symptoms were assessed on a 3-point scale with ‘no more than others’ and ‘a little more than others’ coded as symptom absent and ‘a lot more than others’ coded as symptom present. Although the DAWBA utilises skip rules (omitting questions when screening questions suggest a child is unlikely to have a particular diagnosis), there were no skip rules used in the questions about ADHD symptoms in the ALSPAC cohort. All participants were asked about every ADHD symptom. The wording of the descriptors of the 18 ADHD symptoms are shown in Supplementary Table 1. Most assessed symptoms were worded in the same way at all three ages except for some developmentally appropriate changes (e.g. referring to ‘school’ at ages 7 and 15 and ‘work or study’ at age 25 years), with the exception of two items: (1) *always on the go*, which was assessed based on ‘running/climbing about’ at ages 7 and 15 years, but based on being ‘full of energy and always on the go’ at age 25 years, and (2) *difficulty calming down/relaxing*, which was assessed based on finding it ‘hard to calm down if rushing about’ at ages 7 and 15 years, but based on finding it ‘hard to unwind and relax’ at age 25 years (American Psychiatric Association, 2013). These DAWBA ADHD items showed acceptable measurement invariance across age in this sample (see Supplementary Material).

ADHD impairment was assessed using the five DAWBA impairment and burden items that assess ADHD related impairment in (i) getting on with family and the people they are closest to (e.g. partner), (ii) making and keeping friends, (iii) school/study or work, (iv) leisure activities, and (v) how burdensome these problems are on the individual and others. Items were scored on a 4-point scale (not at all, a little, a medium amount, a great deal) and summed to give a total score (0-15). Comparison of impairment item wording across ages is given in Supplementary Table 2.

### Analyses

Primary analyses were conducted including individuals with parent-rated ADHD data available at all three timepoints (N=2327), using inverse probability weighting (IPW) (Seaman & White, 2013). Weights were derived from a logistic regression analysis of missing ADHD data for those in the core ALSPAC sample (N=13788) for a set of measures assessed in or soon after pregnancy with minimal missingness that showed independent association with missing data (child sex, family home ownership status, maternal depression, age at birth, educationa nd parity). More details about the IPW model are given in the Supplementary Material. Individual symptom frequencies were presented for this sample and the most/least common symptoms described as the three highest/lowest reported symptoms respectively at each age. Mean impairment scores were calculated in the presence of each symptom. Sensitivity analyses were conducted (i) examining symptom frequencies in those who met ADHD diagnostic criteria, (ii) stratifying analyses by sex, and (iii) using different approaches to assess the impact of missing data (complete cases without IPW and including those with any ADHD data) (see Supplementary Material).

## Results

Of the primary sample, 14.8% (95% CI=12.8-17.0) had at least one ADHD symptom at age 7 years, 12.1% (95% CI=10.4-14.0) at age 15 years and 8.5% (95% CI=7.1-10.1) at age 25 years. The mean number of total ADHD symptoms, total hyperactive-impulsive and total inattentive symptoms and associated impairment are shown in Table 1, showing a general decline in symptoms but an increase in associated impairment with age from age 7 to 25 years, particularly for inattentive symptoms.

### Aim 1: Prevalence of Specific ADHD Symptoms

**Figure 1:**
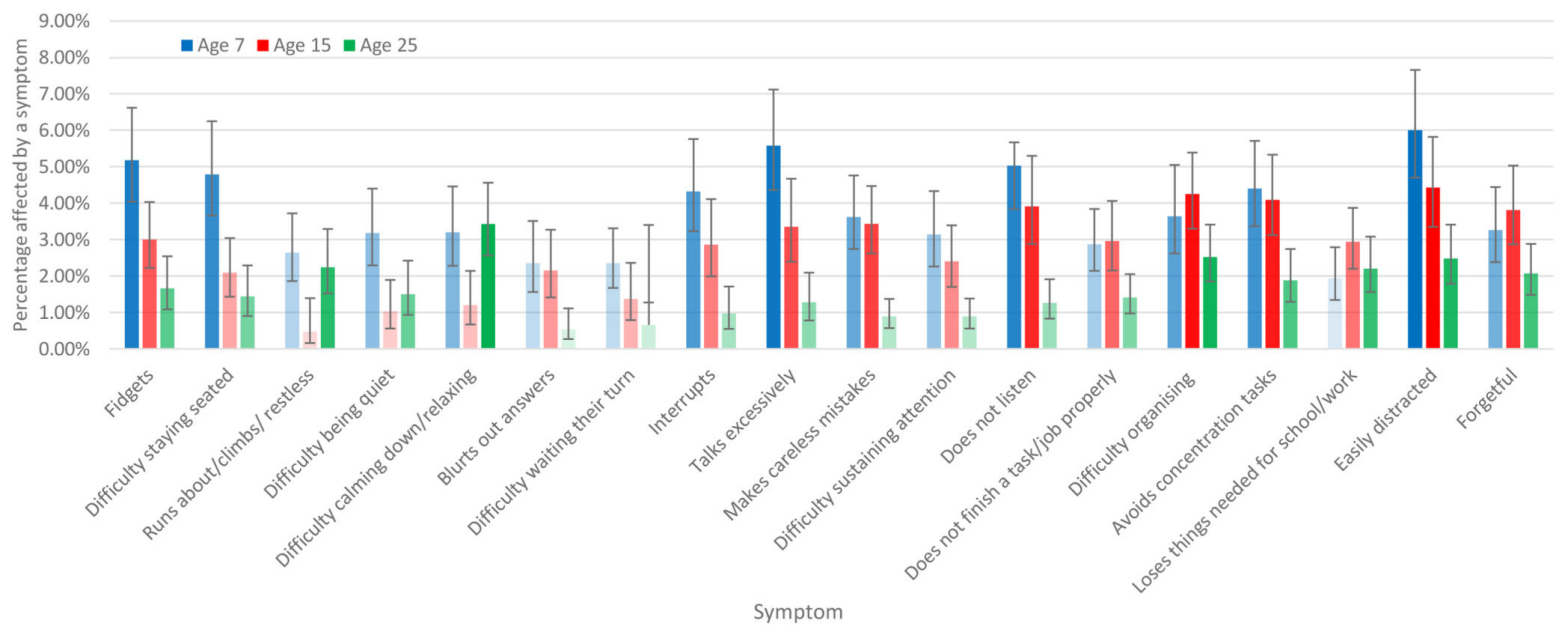
Prevalence of Specific ADHD Symptoms at age 7, 15 and 25 *Note.* Items across age 7, 15 and 25 are the same but wording changes slightly at age 25 to be more appropriate to young adulthood. The first nine symptom descriptors are hyperactive-impulsive symptoms and the latter nine are inattentive symptoms. Colour transparency denotes ranking of symptoms where the higher the rank for symptom prevalence within age, the less transparent the colour. Error bars are the 95% confidence intervals.

ADHD symptom frequencies by age are displayed in Figure 1. Inattentive symptoms showed similar prevalence at ages 7 and 15, with lower prevalence at age 25 years; some of the hyperactive-impulsive symptoms showed the same pattern, but five of them showed a decreased prevalence from age 7 to 15 (items 1-5), with two of these showing a higher prevalence at age 25 years than 15 (runs about/climbs/restless and difficult calming down/relaxing).

The 3 most commonly reported and 3 least commonly reported ADHD symptoms are shown in Table 2. Despite the general trend for symptoms to decline with age, the most common symptoms varied by age with the exception that being *easily distracted* was one of the three most common symptoms at all ages. In childhood, the hyperactive-impulsive items of *fidgets* and *talks excessively* were amongst the most common symptoms. In contrast, the inattentive symptom *difficulty organising* was one of the most common symptoms in both adolescence and young adulthood, along with another inattentive symptom *avoids concentration tasks* in adolescence and the hyperactive-impulsive symptom *difficulty calming down/relaxing* in young adulthood.

### Aim 2: ADHD Symptoms and Associated Impairment

Mean impairment scores for each ADHD symptom by age are shown in Table 3. Mean impairment for hyperactive-impulsive symptoms was similar across age (confidence intervals overlapped), whereas mean impairment for inattentive symptoms was generally higher at age 25 compared to age 7, with intermediate levels at age 15 years (for seven of nine symptoms).

The symptoms associated with the highest mean impairment varied by age with the exception that *difficulty sustaining attention* was one of the most impairing symptoms at all three ages. The symptoms associated with the highest mean impairment included both hyperactive-impulsive and inattentive symptoms at each age. In childhood, *difficulty sustaining attention, does not finish a task/job properly* and *difficulty waiting their turn* were the 3 most impairing symptoms. In adolescence *difficulty sustaining attention, difficulty calming down/relaxing* and *difficulty being quiet* were most impairing, and in young adulthood the three most impairing symptoms were *blurts out answers*, *difficulty waiting their turn* and *difficulty sustaining attention*.

### Sensitivity analyses

Sensitivity analyses found a similar pattern of results examining symptom frequencies in those who met DSM-5 ADHD diagnostic criteria (Supplementary Table 3). Stratifying analyses by sex also showed a similar pattern of results, although there was stronger evidence that some hyperactive-impulsive symptoms were more impairing at age 25 compared to 7 for girls than boys, and that some inattentive symptoms were more impairing at age 25 compared to age 7 for boys than girls (Supplementary Tables 4 and 5). Finally, using different approaches to assess the impact of missing data (Supplementary Tables 6 and 7) also showed a similar pattern of results (see Supplementary Material).

## Discussion

This study aimed to investigate the presentation of specific ADHD symptoms and levels of associated impairment across childhood, adolescence and into young adulthood using prospective longitudinal data from a population cohort. In line with previous work, we found a general trend for ADHD symptom prevalence to decline with age, with hyperactive-impulsive symptoms tending to decline into adolescence and inattentive symptoms remaining more stable in adolescence before declining into young adulthood, when levels of hyperactive-impulsive and inattentive symptoms were similar (Lahey & Willcutt, 2010; Larsson et al., 2011; Holbrook et al., 2016; Todd et al., 2008).

Inspection of specific symptom frequencies found variation in symptom presentation by age. Some symptoms were common at multiple ages including the inattentive symptoms *easily distracted* (common across all three ages) and *difficulty organising* (common in both adolescence and young adulthood). Others were more common at one particular age, for example, the hyperactive-impulsive symptoms *fidgets* and *talks excessively* were two of the most common symptoms in childhood but not in adolescence and young adulthood, and the hyperactive-impulsive symptoms of having *difficulty calming down/relaxing* was one of the most common symptoms in young adulthood, but not at other ages. Indeed, *difficulty calming down/relaxing* was the only symptom which was somewhat more common in young adulthood than at earlier ages. The results also showed that both inattentive and hyperactive-impulsive items were amongst the most common symptoms in childhood and young adulthood, whereas inattentive symptoms showed a pattern of being more commonly reported in adolescence than hyperactive-impulsive symptoms.

It is possible that a combination of changes in the environmental context, expectations and maturational processes contribute to differences in presentation of ADHD symptoms in different developmental periods (Asherson et al., 2012). For example, the difference in prominence of inattentive symptoms at different ages may be due to the differing demands relating to attention across development. Our results suggest inattention symptoms are relatively more common in adolescence than hyperactive-impulsive symptoms. In adolescence greater attention and independence tends to be expected compared to childhood, and school demands are somewhat uniform - compared to in young adulthood when individuals may be more able to select work or study environments that suit their strengths and use compensatory mechanisms which may reduce or mask their symptoms and associated impairment (Kysow et al., 2017; Young et al., 2020). It is not clear why *difficulty calming down/relaxing* showed the unusual pattern of being more commonly reported in young adulthood than at both earlier ages. This could be related to changes in the way this item was assessed in young adulthood compared to childhood and adolescence. In young adulthood the DAWBA item was “is it hard for them to unwind and relax?”, whereas in childhood and adolescence it was “if (s)he is rushing about does (s)he find it hard to calm down when someone asks her/her to do so?”. Changes in the wording of items have large effects on responses (Goodman et al., 2007) and this could have led to a different interpretation of the symptom being addressed. For example, the wording of this item in young adulthood may have been interpreted as relating to stress and anxiety, rather hyperactivity-impulsivity. This highlights the difficulties in designing research to assess continuity and discontinuity in symptoms that may be expressed differently at different developmental periods.

Although the DAWBA, as well as DSM-5, have tried to make the symptom items adaptable for use in young-adulthood, these measures and symptom criteria were designed for use in children. Previous work has suggested that ADHD symptoms can be compared reliably across childhood and adolescence when using the same measure (Murray et al., 2019) and we found acceptable measurement invariance across ages 7, 15 ad 25 years. However, it is possible that young adults who have ADHD-type symptoms could present in a way that is not recognised by these measures. This perhaps could explain the decline in the prevalence of ADHD symptoms into young adulthood.

We also found evidence of developmental differences in ADHD symptom related impairment: while the prevalence of ADHD symptoms declined with age, there was a general trend for the impairment associated with specific symptoms to increase across development – a finding consistent with previous cross-sectional findings (Zoromski et al., 2015). At each developmental period both hyperactive-impulsive and inattentive symptoms were amongst the 3 most impairing symptoms, although there was a general trend for inattentive symptoms to be associated with somewhat higher mean impairment than hyperactive-impulsive symptoms in young adulthood. At all three ages the inattentive symptom *difficulty sustaining attention* was one of the symptoms associated with the highest mean impairment. Other particularly impairing symptoms included the hyperactive-impulsive symptom *difficulty waiting their turn* in both childhood and young adulthood, with *difficulty being quiet* and *difficulty calming down/relaxing* being impairing in adolescence. Unlike at younger ages, in young adulthood *blurts out answers* was one of the symptoms associated with the highest impairment levels. Again, this suggests that specific ADHD symptoms may be particularly impairing to the demands of adolescence compared to childhood or adulthood but also that different symptoms may index particularly high levels of impairment in young adulthood compared to childhood and adolescence. This suggests that current understanding of presentations in childhood/adolescence may not be generalisable to young adulthood. It further suggests that there may be a need for more developmentally sensitive assessments of ADHD symptoms. Although it is important to identify symptoms that are relevant across all developmental stages, prioritising the most impairing symptoms at each age would allow assessments to be more tailored to the developmental stage.

Our findings, therefore, provide preliminary, descriptive evidence that both the common presentations and associated impairment of specific ADHD symptoms differ with age. DSM-5 does acknowledge some developmental differences by (i) providing suggestions of how childhood symptoms may present in adolescence or adulthood (such as *running or climbing about when it is inappropriate* instead presenting as feeling *restless*), and (ii) requiring fewer symptoms to meet diagnostic criteria in adulthood (American Psychiatric Association, 2013). Genetic research also suggests that while there is considerable overlapping genetic liability (approximately 66% shared) between child and adult ADHD (Rovira et al., 2019), there is also likely age-specific genetic liability. Both genetic and environmental factors likely contribute to developmental differences in the presentation of ADHD symptoms, for example exposure to stressful life events or different environmental demands.

Our study suggests benefit in work which continues to investigate ADHD symptoms and impairment into young adulthood, which may aid in a smoother investigation and diagnosis of ADHD in young adulthood and a greater understanding in the prognosis of ADHD from childhood and adolescence into young adulthood. While our descriptive work focussed on individual symptoms, future work could aid clinical detection of ADHD in young adulthood by investigating possible differences in how symptoms cluster together with age, in order to better pinpoint which groups of symptoms may index greater ADHD liability and impairment at different developmental periods. It is also important to note that we examined ADHD symptom presentation across development at a sample-level, rather than examining within-person changes over time. Our results therefore provide insight into the presentation of ADHD at different ages, but do not capture how an individual’s symptom presentation may change with development - for example a similar symptom frequency at different ages could result from different individuals within the sample presenting with this symptom at these time-points. Investigating within-person changes and heterogeneity in symptom presentation across development would be an interesting area for further research.

Findings of this study should be considered in light of a number of limitations. First, we used parent ratings of ADHD symptoms at all three ages to enable consistent reporting and rigorous comparisons across development. Research shows parents to be good informants of ADHD symptoms in childhood and adolescence (Kuhn et al., 2017), and although work suggests parents are still useful informants in young adulthood (Barkley et al., 2002) this is not in line with clinical practice in adulthood when self-report would be used. Recent research in this sample suggests validity and utility of parent-ratings of ADHD symptoms (Riglin et al., 2020) but this needs further validation and may still lead to different findings compared to self-reports, as parents would be observing symptoms whereas the individual would be experiencing the symptoms (across all three developmental periods in our study). Similarly, teacher reports are often used in childhood which may also show different results (Narad et al., 2015).

Second, like other prospective cohorts, ALSPAC suffers from non-random attrition whereby those at greater risk of psychopathology are more likely to drop out of the study (Boyd et al., 2013; Taylor et al., 2018); estimated prevalence rates and associated impairment are therefore likely underestimates of the population mean (Caye et al., 2016) although we used inverse probability weighting to try and minimise the impact of missing data and findings were consistent across different approaches to missing data. The findings from this population sample also may not generalize to clinical samples, although sensitivity analyses found a similar pattern of results for those meeting diagnostic criteria for ADHD. Nevertheless, clinical samples may show different patterns due to higher rates of comorbidities which could exacerbate both the presentation of their symptoms and impairment on social, occupational and academic functioning (Asherson et al., 2012). The use of a population sample also limits sample size for those with ADHD symptoms which limits power, reflected in our wide confidence intervals.

In summary, this study investigated the prevalence of specific ADHD symptoms and associated impairment using prospective longitudinal data from a population cohort across childhood, adolescence and into young adulthood. We found evidence of varying ADHD symptom presentation across different developmental periods, with different hyperactive-impulsive and inattentive symptoms more common at different ages. We also found a trend for the presence of symptoms to be associated with higher levels of impairment as age increased and while some symptoms were associated with high levels of impairment at multiple ages, some symptoms appeared to be particularly impairing in young adulthood. This suggests that the presentation of ADHD and its associated impairment changes with development, and that different symptoms may be more informative at different ages.

## Supplementary Material

Supplementary Material

## Figures and Tables

**Table 1:** Mean number of ADHD symptoms and impairment scores at ages 7, 15 and 25 years

	Mean number of symptoms (95% CI)			Mean total impairment (95% CI)		
	Total ADHD symptoms	Hyperactive -impulsive symptoms	Inattentive symptoms	Any ADHD symptom(s)	Hyperactive -impulsive symptom(s)	Inattentive symptom(s)
Age 7	0.67	0.34	0.34	4.23	4.61	4.77
	(0.55-0.80)	(0.26-0.41)	(0.27-0.41)	(3.63-4.84)	(3.91-5.31)	(4.06-5.49)
Age 15	0.50	0.18	0.32	5.99	6.22	6.42
	(0.39-0.61)	(0.12-0.23)	(0.25-0.39)	(5.08-6.89)	(4.98-7.45)	(5.44-7.40)
Age 25	0.29	0.14	0.16	6.44	6.21	8.21
	(0.23-0.36)	(0.10-0.18)	(0.12-0.20)	(5.56-7.32)	(5.08-7.34)	(7.24-9.17)

*Note.* The maximum number of ADHD symptoms possible is 18 (9 hyperactive-impulsive and 9 inattentive). The maximum impairment score possible is 15.

**Table 2:** Most commonly and least commonly reported ADHD symptoms at ages 7, 15 and 25

	Age 7	Age 15	Age 25
Three most reported symptoms	*1. Fidgets*	*1. Easily distracted*	*1. Difficulty calming down/relaxing*
	*2. Talks excessively*	*2. Difficulty organising*	*2. Difficulty organising*
	*3. Easily Distracted*	*3. Avoids concentration tasks*	*3. Easily distracted*
Three least reported symptoms	*1. Loses things needed for school/work*	*1. Runs about/climbs/restless*	*1. Blurts out answers*
	*2. Difficulty waiting their turn*	*2. Difficulty being quiet*	*2. Difficulty waiting their turn*
	*3. Blurts out answers*	*3. Difficulty calming down/relaxing*	*3. Difficulty sustaining attention*

*Note.* The most and least commonly reported symptoms are symptoms which were the three most reported and three least reported symptoms, respectively.

**Table 3:** Mean Impairment Score for Each ADHD Symptom at Ages 7, 15 and 25 years

	Mean impairment score (95% CI)		
	Symptom present at age 7	Symptom present at age 15	Symptom present at age 25
*Hyperactive-impulsive symptoms*			
1: Fidgets	5.59 (4.50-6.69)	6.90 (4.92-8.88)	8.33 (6.41-10.25)
2: Difficulty staying seated	5.27 (4.10-6.44)	7.45 (4.99-9.91)	8.99 (7.16-10.83)
3: Runs about/climbs/restless	6.54 (4.94-8.14)	7.67 (-0.95-16.29)	5.65 (3.71-7.59)
4: Difficulty being quiet	5.93 (4.39-7.46)	8.78* (5.04-12.53)	9.34 (7.23-11.44)
5: Difficulty calming down/relaxing	5.79 (4.11-7.48)	8.95* (5.98-11.92)	7.78 (6.22-9.34)
6: Blurts out answers	5.04 (3.44-6.65)	6.69 (4.24-9.14)	11.06* (8.90-13.21)
7: Difficulty waiting their turn	7.47* (6.27-8.68)	7.35 (3.59-11.10)	10.78* (7.89-13.67)
8: Interrupts	5.24 (4.04-6.44)	6.71 (4.70-8.71)	9.79 (7.44-12.14)
9: Talks excessively	4.98 (3.94-6.03)	6.78 (5.06-8.49)	8.55 (5.84-11.26)
*Inattentive symptoms*			
10: Makes careless mistakes	6.29 (5.35-7.23)	8.26 (7.12-9.40)	9.39 (7.44-11.33)
11: Difficulty sustaining attention	6.97* (5.95-7.98)	9.05* (7.65-10.44)	10.52* (8.40-12.65)
12: Does not listen	6.08 (4.97-7.20)	6.91 (5.33-8.48)	9.56 (7.05-12.06)
13: Does not finish a task/job properly	6.87* (5.88-7.86)	8.16 (6.76-9.57)	9.01 (6.90-11.12)
14: Difficulty organising	5.54 (4.29-6.78)	7.53 (6.34-8.72)	8.92 (7.87-9.97)
15: Avoids concentration tasks	5.42 (4.56-6.29)	7.49 (5.97-9.01)	9.34 (7.75-10.94)
16: Loses things needed for school/work	6.18 (4.92-7.44)	6.90 (5.31-8.49)	9.24 (7.97-10.51)
17: Easily distracted	5.24 (4.33-6.15)	7.05 (5.57-8.54)	9.41 (8.26-10.56)
18: Forgetful	5.52 (4.33-6.70)	7.17 (5.73-8.60)	8.26 (7.00-9.52)

*Note.* Maximum mean impairment score is 15. The asterisk (*) denotes the three symptoms associated with the highest impairment score at each age group.

## References

[R1] American Psychiatric Association (2013). Attention-Deficit/Hyperactivity Disorder (ADHD). Diagnostic and Statistical Manual of Mental Disorders.

[R2] Asherson P, Akehurst R, Kooij JJ, Huss M, Beusterien K, Sasané R, Gholizadeh S, Hodgkins P (2012). Under diagnosis of adult ADHD: cultural influences and societal burden. J Atten Disord.

[R3] Barkley RA (2011). Barkley Adult ADHD Rating Scale-IV (BAARS-IV).

[R4] Barkley RA, Fischer M, Smallish L, fletcher K (2002). The persistence of attention-deficit/hyperactivity disorder into young adulthood as a function of reporting source and definition of disorder. J Abnorm Psychol.

[R5] Boyd A, Golding J, Macleod J, Lawlor DA, Fraser A, Henderson J, Molloy L, Ness A, Ring S, Davey smith G (2013). Cohort profile: the ‘children of the 90s’—the index offspring of the Avon Longitudinal Study of Parents and Children. International journal of epidemiology.

[R6] Caye A, Swanson J, Thapar A, Sibley M, Arseneault L, Hechtman L, Arnold LE, Niclasen J, Moffitt T, Rohde LA (2016). Life Span Studies of ADHD—Conceptual Challenges and Predictors of Persistence and Outcome. Current Psychiatry Reports.

[R7] Faraone SV, Biederman J, Mick E (2006). The age-dependent decline of attention deficit hyperactivity disorder: a meta-analysis of follow-up studies. Psychological medicine.

[R8] Fraser A, Macdonald-Wallis C, Tilling K, Boyd A, Golding J, Davey Smith G, Henderson J, Macleod J, Molloy L, Ness A, Ring S (2013). Cohort Profile: the Avon Longitudinal Study of Parents and Children: ALSPAC mothers cohort. Int J Epidemiol.

[R9] Goodman A, Heiervang E, Collishaw S, Goodman R (2011). The ‘DAWBA bands’ as an ordered-categorical measure of child mental health: description and validation in British and Norwegian samples. Soc Psychiatry Psychiatr Epidemiol.

[R10] Goodman R, Ford T, Richards H, Gatward R, Meltzer H (2000). The Development and WellBeing Assessment: description and initial validation of an integrated assessment of child and adolescent psychopathology. Journal of child psychology and psychiatry, and allied disciplines.

[R11] Goodman R, Iervolino AC, Collishaw S, Pickles A, Maughan B (2007). Seemingly minor changes to a questionnaire can make a big difference to mean scores: a cautionary tale. Social psychiatry and psychiatric epidemiology.

[R12] Caye A, Swanson J, Thapar A, Sibley M, Arseneault L, Hechtman L, Arnold LE, Niclasen J, Moffitt T, Rohde LA (2016). Life Span Studies of ADHD—Conceptual Challenges and Predictors of Persistence and Outcome. Current Psychiatry Reports.

[R13] Harris PA, Taylor R, Minor BL, Elliott V, Fernandez M, O’Neal L, McLeod L, Delacqua G, Delacqua F, Kirby J, Duda SN (2019). The REDCap consortium: Building an international community of software platform partners. J Biomed Inform.

[R14] Harris PA, Taylor R, Thielke R, Payne J, Gonzalez N, Conde JG (2009). Research electronic data capture (REDCap)--a metadata-driven methodology and workflow process for providing translational research informatics support. J Biomed Inform.

[R15] Murray AL, Obsuth I, Eisner M, Ribeaud D (2019). Evaluating Longitudinal Invariance in Dimensions of Mental Health Across Adolescence: An Analysis of the Social Behavior Questionnaire. Assessment.

[R16] Seaman SR, White IR (2013). Review of inverse probability weighting for dealing with missing data. Stat Methods Med Res.

[R17] Holbrook JR, Cuffe SP, Cai B, Visser SN, Forthofer MS, Bottai M, Ortaglia A, McKeown RE (2016). Persistence of Parent-Reported ADHD Symptoms From Childhood Through Adolescence in a Community Sample. J Atten Disord.

[R18] Kirova A-M, Kelberman C, Storch B, DiSalvo M, Woodworth KY, Faraone SV, Biederman J (2019). Are subsyndromal manifestations of attention deficit hyperactivity disorder morbid in children? A systematic qualitative review of the literature with meta-analysis. Psychiatry Research.

[R19] Kuhn C, Aebi M, Jakobsen H, Banaschewski T, Poustka L, Grimmer Y, Goodman R, Steinhausen H-C (2017). Effective Mental Health Screening in Adolescents: Should We Collect Data from Youth, Parents or Both?. Child Psychiatry & Human Development.

[R20] Kysow K, Park J, Johnston C (2017). The use of compensatory strategies in adults with ADHD symptoms. ADHD Attention Deficit and Hyperactivity Disorders.

[R21] Lahey BB, Willcutt EG (2010). Predictive validity of a continuous alternative to nominal subtypes of attention-deficit/hyperactivity disorder for DSM-V. Journal of clinical child and adolescent psychology : the official journal for the Society of Clinical Child and Adolescent Psychology, American Psychological Association, Division 53.

[R22] Larsson H, Dilshad R, Lichtenstein P, Barker ED (2011). Developmental trajectories of DSM-IV symptoms of attention-deficit/hyperactivity disorder: genetic effects, family risk and associated psychopathology. Journal of Child Psychology and Psychiatry.

[R23] Narad ME, Garner AA, Peugh JL, Tamm L, Antonini TN, Kingery KM, Simon JO, Epstein JN (2015). Parent-teacher agreement on ADHD symptoms across development. Psychological assessment.

[R24] National Institute of Health and Clinical Excellence (2018). Attention deficit hyperactivity disorder: diagnosis and management.

[R25] Northstone K, Lewcock M, Groom A, Boyd A, Macleod J, Timpson N, wells N (2019). The Avon Longitudinal Study of Parents and Children (ALSPAC): an update on the enrolled sample of index children in 2019. Wellcome Open Res.

[R26] Polanczyk GV, Salum GA, Sugaya LS, Caye A, Rohde LA (2015). Annual Research Review: A meta-analysis of the worldwide prevalence of mental disorders in children and adolescents. Journal of Child Psychology and Psychiatry.

[R27] Riglin L, Leppert B, Langley K, Thapar AK, O’Donovan MC, DaveySmith G, Stergiakouli E, Tilling K, Thapar A (2020). Investigating attention-deficit hyperactivity disorder and autism spectrum disorder traits in the general population: What happens in adult life?. Journal of Child Psychology and Psychiatry.

[R28] Rovira P, Demontis D, Sánchez-Mora C, Zayats T, Klein M, Mota NR, Weber H, Garcia-Martínez I, Pagerols M, Vilar L, Arribas L (2019). Shared genetic background between children and adults with attention deficit/hyperactivity disorder. bioRxiv.

[R29] Taylor AE, Jones HJ, Sallis H, Euesden J, Stergiakouli E, Davies NM, Zammit S, Lawlor DA, Munafò MR, Davey Smith G, Tilling K (2018). Exploring the association of genetic factors with participation in the Avon Longitudinal Study of Parents and Children. International journal of epidemiology.

[R30] Thapar A, Gooper M (2016). Attention deficit hyperactivity disorder. Lancet.

[R31] Thapar A, Riglin L (2020). The importance of a developmental perspective in Psychiatry: what do recent genetic-epidemiological findings show?. Molecular Psychiatry.

[R32] Todd RD, Huang H, Todorov AA, Neuman RJ, Reiersen AM, Henderson CA, Reich WC (2008). Predictors of stability of attention-deficit/hyperactivity disorder subtypes from childhood to young adulthood. Journal of the American Academy of Child & Adolescent Psychiatry.

[R33] Young S, Hollingdale J, Absoud M, Bolton P, Branney P, Colley W, Craze E, Dave M, Deeley Q, Farrag E, Gudjonsson G (2020). Guidance for identification and treatment of individuals with attention deficit/hyperactivity disorder and autism spectrum disorder based upon expert consensus. BMcmedicine.

[R34] Zoromski AK, Owens JS, Evans SW, Brady CE (2015). Identifying ADHD Symptoms Most Associated with Impairment in Early Childhood, Middle Childhood, and Adolescence Using Teacher Report. J Abnorm Child Psychol.

