# Supplementary material for: Exploring ADHD Symptoms and Associated Impairment Across Development
Source: J Atten Disord. Author manuscript; Available in PMC 2022 Apr 1. (PMC7612406; doi:10.1177/10870547211025612)
Supplement: Supplementary Material [file EMS128746-supplement-Supplementary_Material.pdf]

Supplementary Material

Measurement invariance

We assessed measurement invariance by age using structural equation modelling to a latent structure with one inattention factor and one hyperactivity-impulsivity factor, each indexed by the relevant nine binary ADHD symptoms. In-line with recommendations (Putnick & Bornstein, 2016), we evaluated increasingly stringent types of measurement invariance: (i) configural invariance (same pattern of free and fixed loadings across age), (ii) metric invariance (similar degree of factor loadings across age), (iii) scalar invariance (similar items thresholds across age), and (iv) residual invariance (similar item residuals across age). Due to the use of binary indicators (symptoms), configural and metric invariance could not be assessed without fixing residuals across time (i.e. fixed at 1), because freeing thresholds and residuals leads to model nonidentification. Residual variances were therefore fixed to be equal when testing configural and metric invariance, which means that the four models are not all nested. Models were fit in Mplus (Muthén & Muthén, 1998-2012) using full information maximum likelihood (FIML) for those with any available symptom data (N=8805) with weighted least square parameter estimates (WLSMV) using the using the theta parameterization to enable the modelling of residual variances. Model fit was assessed by fit indices including the comparative fit index (CFI), root-mean-square error of approximation (RMSEA) and standardized root mean squared residual (SRMR), for which values of ≥0.95, ≤0.06 and ≤0.08 are generally considered good fit (Hu & Bentler, 1999). Model fit indices are shown in Supplementary Table 8: the initial two-factor structure, in which factors loadings and thresholds were free across age (configural model A) showed good model fit and fixing factor loadings (model B) retained good model fit (ΔCFI<0.01) (Cheung & Rensvold, 2002; Putnick & Bornstein, 2016). A model of strict measurement invariance (residuals invariance model D) in which factor loadings, thresholds and residuals were fixed also showed good model fit, without strong evidence of poorer fit compared to models either where residuals were not fixed across age (model C) or where thresholds were not fixed across age (model

B). These models provide evidence of reasonable measurement invariance of the DAWBA ADHD items across ages 7, 15 and 25 years.

Inverse probability weighting

The primary sample included individuals with parent-rated ADHD data available at ages 7, 15 and 25 years (N=2,327). Inverse probability weighting (IPW) was used to try to minimise the impact of missing ADHD data (Seaman & White, 2013). IPW has been recommended over alternative methods such as multiple imputation in situations where whole blocks of data are missing for a large proportion of individuals (Seaman et al., 2012). Weights were derived from a logistic regression analysis of missing ADHD data for those in the full 'core' ALSPAC sample (N= 13,788) for a set of measures assessed in or soon after pregnancy with minimal missingness that were that showed independent association with missing data (see Supplementary Table 9) and weights were stabilized whereby the numerator was the probability of inclusions in the sample (i.e. 2327/13788) (Sayon-Orea et al., 2020). The Hosmer-Lemeshow test was used assess the fit of the missingness model; results did not indicate poor fit (Hosmer-Lemeshow $\chi 2(8)=10.76$, $p=0.22$). For the included sample (N=2,327) weights ranged from 0.35 to 8.59. Weights were used to estimate proportions to assess symptom frequencies and to estimate mean impairment scores for those with observed symptoms.

ADHD diagnoses sensitivity analyses

Sensitivity analyses were conducted to examine symptom frequencies in those who met ADHD diagnostic criteria. DSM-5 ADHD diagnoses were generated based on DAWBA computer algorithms (Goodman et al., 2011) at ages 7 and 15 years and diagnosis based on the Barkley Adult ADHD Rating Scale (BAARS-IV) (Barkley, 2011) at age 25 years. Analyses found a similar pattern of results in those meeting ADHD diagnostic criteria as those found in the whole sample, with many of the most commonly reported and least commonly reported ADHD symptoms consistent: see Supplementary Table 3. However, there were some minor differences including that 'difficulty waiting their turn' at

age 7 was recorded as one of the 3 least commonly reported symptoms in the whole sample, yet it was 7th most commonly reported in those meeting diagnostic criteria for ADHD. The symptom 'avoids concentration tasks' at age 15 was one of 3 most commonly reported symptoms in the whole sample, but only the 10th most commonly reported symptom in those meeting diagnostic criteria for ADHD and, the symptom 'difficulty calming down/relaxing' at age 25 was the most commonly reported symptom in the whole sample, but only 10th most common in those meeting criteria for ADHD diagnosis.

Sex specific sensitivity analyses

Sensitivity analyses examined ADHD symptom frequencies and associated mean impairment score separately for males and females (Supplementary Tables 4 and 5 respectively). Results showed a similar pattern of results, although there were some differences in the most commonly ranked symptoms for boys and girls. There was also stronger evidence that some hyperactive-impulsive symptoms were more impairing at age 25 compared to age 7 for girls than boys (difficulty staying seated, blurting out answer and interrupts), and that some inattentive symptoms were more impairing at age 25 compared to age 7 for boys than girls (does not listen, does not finish task/job properly, difficulty sustaining attention).

Missing data sensitivity analyses

Sensitivity analyses were conducted (i) using 'complete case analyses' (i.e. N=2327 without IPW) and (ii) including anyone with any ADHD data (maximum N=8805). These approaches showed a a similar pattern of results to the primary (IPW) analyses (Supplementary Tables 6 and 7).

Supplementary Table 1

Comparison of the Descriptions of the DSM-5 ADHD Symptom Criteria With our Study and the DAWBA Questionnaire at Ages 7, 15 and 25 years

	DSM-5 Criteria (American Psychiatric Association, 2013)	Symptom description used in study	Age 7	Age 15	Age 25
HAI 1	Often fidgets with or taps hands or feet or squirms in seat.	Fidgets	Does s/he often fidget?	Does s/he often fidget?	Does s/he often fidget?
HAI 2	Often leaves seat in situations when remaining seated is expected (e.g., leaves his or her place in the classroom, in the office or other workplace, or in other situations that require remaining in place).	Difficulty staying seated	Is it hard for him/her to stay sitting down for long?	Is it hard for him/her to stay sitting down for long?	Is it hard for him/her to stay sitting down for long?
HAI 3	Often runs about or climbs in situations where it is inappropriate. (Note: In adolescents or adults, may be limited to feeling restless.)	Runs about/climbs/ restless	Does s/he run or climb about when she shouldn't?	Does s/he run or climb about when s/he shouldn't?	Is s/he too full of energy and always on the go?
HAI 4	Often unable to play or engage in leisure activities quietly.	Difficulty being quiet	Does s/he find it hard to play or take part in other leisure activities without making a noise?	Does s/he find it hard to play or take part in other leisure activities without making a lot of noise?	Does s/he find it difficult to be quiet?
HAI 5	Is often "on the go," acting as if "driven by a motor" (e.g., is unable to be or uncomfortable being still for extended time, as in restaurants, meetings; may be experienced by others as being restless or difficult to keep up with).	Difficulty calming down/relaxing	If s/he is rushing about does s/he find it hard to calm down when someone asks her to do so?	If s/he is rushing about, does s/he find it hard to calm down when someone asks him/her to?	Is it hard for him/her to unwind and relax?
HAI 6	Often blurts out an answer before a question has been completed (e.g., completes people's sentences; cannot wait for turn in conversation).	Blurts out answers	Does s/he often blurt out an answer before s/he has heard the question properly?	Does s/he often blurt out an answer before s/he had heard the question properly?	Does s/he often blurt out an answer before s/he has heard the question properly or finish other people's questions for them?
HAI 7	Often has difficulty waiting his or her turn (e.g., while waiting in line).	Difficulty waiting their turn	Is it hard for him/her to wait her turn?	Is it hard for him/her to wait his/her turn?	Is it hard for him/her to wait his/her turn?
HAI 8	Often interrupts or intrudes on others (e.g., butts into conversations, games, or activities; may start using other people's things without asking or receiving permission; for adolescents and adults, may intrude into or take over what others are doing).	Interrupts	Does s/he often butt in on other people/s conversation or games?	Does s/he often butt in on other people/s conversations or games?	Does s/he often interrupt other people when they are busy?
HAI 9	Often talks excessively.	Talks excessively	Does s/he often go on talking even if s/he has been asked to stop or no one is listening?	Does s/he often go on talking even if s/he has been asked to stop, or if no one is listening?	Does s/he often go on talking even if s/he have been asked to stop or no one is listening?
IA 1	Often fails to give close attention to details or makes careless mistakes in schoolwork, at work, or during other activities (e.g., overlooks or misses details, work is inaccurate).	Makes careless mistakes	Does s/he often make careless mistakes or fail to pay attention to what s/he is supposed to be doing?	Does s/he often make careless mistakes or fail to pay attention to what s/he is supposed to be doing?	Does s/he often make careless mistakes or fail to pay attention to what s/he is supposed to be doing?

IA 2	Often has difficulty sustaining attention in tasks or play activities (e.g., has difficulty remaining focused during lectures, conversations, or lengthy reading).	Difficulty sustaining attention	Does s/he often seem to lose interest in what s/he is doing?	Does s/he often seem to lose interest in what s/he is doing?	Does s/he often seem to lose interest in what s/he is doing?
IA 3	Often does not seem to listen when spoken to directly (e.g., mind seems elsewhere, even in the absence of any obvious distraction).	Does not listen	Does s/he often not listen to what people are saying to him/her?	Does s/he often no listen to what people are saying to him/her?	Does s/he often not listen to what people are saying to him/her?
IA 4	Often does not follow through on instructions and fails to finish schoolwork, chores, or duties in the workplace (e.g., starts tasks but quickly loses focus and is easily side-tracked).	Does not finish a task/job properly	Does s/he often not finish a job properly?	Does s/he often not finish a job properly?	Does s/he often not finish a job properly?
IA 5	Often has difficulty organizing tasks and activities (e.g., difficulty managing sequential tasks; difficulty keeping materials and belongings in order; messy, disorganized work; has poor time management; fails to meet deadlines).	Difficulty organising	Is it often hard for him/ her to get herself organised to do something?	Is it hard for him/her to get himself/herself organised to do something?	Is it often hard for him/her to get himself/herself organised to do something?
IA 6	Often avoids, dislikes, or is reluctant to engage in tasks that require sustained mental effort (e.g., schoolwork or homework; for older adolescents and adults, preparing reports, completing forms, reviewing lengthy papers).	Avoids concentration tasks	Does s/he often try to get out of things s/he would have to think about, such as homework?	Does s/he often try to get out of things s/he would have to think about, such as homework?	Does s/he often try to get out of things s/he would have to think about?
IA 7	Often loses things necessary for tasks or activities (e.g., school materials, pencils, books, tools, wallets, keys, paperwork, eyeglasses, mobile telephones).	Loses things needed for school/work	Does s/he often lose things s/he needs for school or PE?	Does s/he often lose things s/he needs for school or games?	Does s/he often lose things s/he needs for work or study?
IA 8	Is often easily distracted by extraneous stimuli (for older adolescents and adults, may include unrelated thoughts).	Easily distracted	Is s/he easily distracted?	Is s/he easily distracted?	Is s/he easily distracted?
IA 9	Is often forgetful in daily activities (e.g., doing chores, running errands; for older adolescents and adults, returning calls, paying bills, keeping appointments).	Forgetful	Is s/he often forgetful?	Is s/he often forgetful?	Is s/he often forgetful?

Note. HAI= hyperactive-impulsive symptoms and IA= inattentive symptoms

Supplementary Table 2.

Comparison of the Descriptions of the DSM-5 ADHD Impairment Criteria With our Study and the DAWBA Questionnaire at Ages 7, 15 and 25

	DSM-5 Criteria (American Psychiatric Association, 2013)	Suggestion	Age 7	Age 15	Age 25
Impairment 1	Symptoms interfere with, or reduce the quality of, social, academic, or occupational functioning	Family/partner	How well s/he gets on with you and rest of family	How well s/he gets on with you and rest of family	How well s/he get on with people s/he is closest to (e.g. family, partner)
Impairment 2		Friends	Making and keeping friends	Making and keeping friends	Making and keeping friends
Impairment 3		School/work	Learning or school work	Learning or class work	Work or study
Impairment 4		Leisure	Playing, hobbies, sports or other leisure activities	Playing, hobbies, sports or other leisure activities	Hobbies, sports or other leisure activities
Impairment 5		Burden on self/others	Have these problems put a burden on you or the family as a whole?	Have these difficulties with activity or concentration put a burden on you or the family as a whole?	Have these problems put a burden on you or others?

Supplementary Table 3

Prevalence of Specific ADHD Symptoms at age, 7, 15 and 25 in those who Meet ADHD Diagnosis with 95% confidence intervals

	In those who meet ADHD Diagnosis			Primary sample ranking for comparison		
	Age 7	Age 15	Age 25	Age 7	Age 15	Age 25
Hyperactive-impulsive symptoms						
1: Fidgets	(3rd)	(8th)	46.2% (24.1-69.9), 7th	3rd	8th	8th
2: Difficulty staying seated	(4th)	(12th)	29.3% (12.0-55.8), 14th	5th	14th	10th
3: Runs about/climbs/ restless	58.4% (31.4-81.2), 15th	(18th)	21.3% (6.7-50.4), 16th	15th	18th	4th
4: Difficulty being quiet	69.9% (41.7-88.3), 13th	52.7% (19.4-83.7), 15th	32.7% (14.4-58.5), 13th	12th	17th	9th
5: Difficulty calming down/relaxing	74.7% (46.4-91.0), 10th	55.9% (22.3-84.8), 14th	42.9% (21.8-67.0), 10th	11th	16th	1st
6: Blurts out answers	29.9% (12.3-56.5), 18th	62.3% (26.2-88.5), 13th	(18th)	16th	13th	18th
7: Difficulty waiting their turn	(7th)	52.3% (19.1-83.5), 16th	(17th)	17th	15th	17th
8: Interrupts	75.0% (47.6-90.8), 9th	(6th)	21.4% (9.2-42.3), 15th	7th	11th	14th
9: Talks excessively	(6th)	(11th)	34.8% (16.0-59.9), 12th	2nd	7th	12th
Inattentive symptoms						
10: Makes careless mistakes	(5th)	(7th)	35.0% (16.9-58.8), 11th	8th	6th	15th
11: Difficulty sustaining attention	80.6% (50.9-94.3), 8th	(2nd)	43.9% (22.9-67.4), 9th	13th	12th	16th
12: Does not listen	(2nd)	(5th)	47.8% (25.5-71.0), 6th	4th	4th	13th
13: Does not finish a task/job properly	73.7% (35.2-93.5), 11th	(4th)	51.6% (28.1-74.4) 5th	14th	9th	11th
14: Difficulty organising	72.0% (43.8-89.5), 12th	(3rd)	62.8% (35.4-83.8, 3rd	9th	2nd	2nd
15: Avoids concentration tasks	59.0% (29.2-83.3), 14th	(10th)	58.6% (32.8-80.4), 4th	6th	3rd	7th
16: Loses things needed for school/work	47.7% (22.3-74.3), 17th	37.9% (12.5-72.3), 17th	65.3% (39.1-84.7), 2nd	18th	10th	5th
17: Easily distracted	100%, 1st	(1st)	79.4% (47.2-94.3), 1st	1st	1st	3rd
18: Forgetful	52.3% (25.1-78.1), 16th	(9th)	45.5% (23.9-68.8), 8th	10th	5th	6th

Note. N=23 with diagnosis at age 7, N=14 at age 15 and N=26 at age 25. Proportions derived using inverse probability weighting. 95% confidence intervals in parentheses. Items across age 7, 15 and 25 are the same but wording changes slightly at age 25 to be more appropriate to young adulthood. Percentages not reported where estimated N<5.

Supplementary Table 4

Prevalence of Specific ADHD Symptoms at age, 7, 15 and 25 separately for males and females

	Males			Females		
	Age 7	Age 15	Age 25	Age 7	Age 15	Age 25
Hyperactive-impulsive symptoms						
1: Fidgets	6.8%* (5.0-9.2)	4.1% (2.8-5.8)	2.0% (1.2-3.5)	3.6% (2.3-5.5)	1.9% (1.2-3.3)	1.3% (0.6-2.6)
2: Difficulty staying seated	6.7% (4.9-9.2)	2.6% (1.6-4.3)	1.8% (1.0-3.2)	2.9% (1.7-4.8)	1.6% (0.9-2.7)	1.1% (0.5-2.3)
3: Runs about/climbs/ restless	3.1% (2.0-4.9)	0.9% (0.3-2.8)	2.1% (1.1-3.9)	2.1% (1.2-3.7)	-	2.4% (1.5-3.8)
4: Difficulty being quiet	4.1% (2.7-6.3)	1.3% (0.5-3.0)	1.8% (0.9-3.4)	2.3% (1.3-3.8)	0.8% (0.4-1.7)	1.2% (0.6-2.4)
5: Difficulty calming down/relaxing	4.1% (2.7-6.2)	1.8% (0.9-3.6)	3.9%* (2.6-5.6)	2.3% (1.3-4.1)	0.6% (0.2-1.6)	3.0%* (1.9-4.6)
6: Blurts out answers	2.9% (1.7-4.8)	3.4% (2.1-5.5)	0.6% (0.3-1.2)	1.8% (0.9-3.5)	0.9% (0.5-1.9)	0.5 (0.2-1.8)
7: Difficulty waiting their turn	3.3% (2.2-5.1)	1.9% (1.0-3.8)	0.5% (0.2-1.2)	1.4% (0.8-2.4)	0.8% (0.3-1.9)	0.8% (0.3-2.0)
8: Interrupts	5.4% (3.8-7.7)	4.2% (2.6-6.5)	1.1% (0.5-2.2)	3.2% (2.0-5.2)	1.6% (0.9-2.9)	0.9% (0.3-2.2)
9: Talks excessively	7.4%* (5.4-10.0)	4.2% (2.7-6.3)	1.3% (0.7-2.4)	3.8%* (2.6-5.7)	2.6%* (1.5-4.4)	1.3% (0.6-2.7)
Inattentive symptoms						
10: Makes careless mistakes	5.2% (3.7-7.1)	4.4% (3.1-6.1)	0.9% (0.6-1.7)	2.1% (1.3-3.5)	2.5%* (1.6-3.9)	0.8% (0.4-1.6)
11: Difficulty sustaining attention	4.1% (2.7-6.1)	3.7% (2.5-5.6)	1.0% (0.5-1.7)	2.2% (1.3-3.7)	1.1% (0.6-2.1)	0.9% (0.4-1.6)
12: Does not listen	6.3% (4.5-8.7)	6.0% (4.1-8.6)	1.1% (0.6-2.1)	3.8%* (2.4-6.1)	1.9% (1.2-3.0)	1.4% (0.8-2.4)
13: Does not finish a task/job properly	3.6% (2.5-5.3)	4.2% (2.8-6.2)	1.6% (1.0-2.6)	2.1% (1.3-3.4)	1.8% (1.1-2.9)	1.2% (0.7-2.2)
14: Difficulty organising	4.7% (3.3-6.7)	6.7%* (5.0-8.8)	3.1%* (2.0-4.6)	2.6% (1.3-5.0)	1.9% (1.2-2.8)	2.0% (1.3-3.1)
15: Avoids concentration tasks	5.1% (3.6-7.1)	6.3%* (4.6-8.6)	2.1% (1.2-3.6)	3.7% (2.5-5.6)	2.0% (1.2-3.2)	1.7% (1.0-2.7)
16: Loses things needed for school/work	2.5% (1.6-4.0)	4.5% (3.3-6.3)	2.4%* (1.5-3.7)	1.4% (0.8-2.5)	1.4% (0.8-2.2)	2.0% (1.2-3.4)
17: Easily distracted	7.6%* (5.7-10.1)	6.9%* (5.0-9.5)	2.3% (1.4-3.8)	4.4%* (2.8-6.9)	2.0%* (1.2-3.2)	2.6%* (1.7-4.0)
18: Forgetful	4.7% (3.3-6.6)	6.2% (4.4-8.5)	1.9% (1.2-3.1)	1.9% (1.0-3.5)	1.5% (0.9-2.4)	2.2%* (1.4-3.5)

Note. N=1065 males and N=1262 females. Proportions derived using inverse probability weighting. 95% confidence intervals in parentheses. Items across age 7, 15 and 25 are the same but wording changes slightly at age 25 to be more appropriate to young adulthood. The asterisk (*) denotes the three most common symptoms at each age group. Percentages not reported where estimated N<5.

Supplementary Table 5

Mean Impairment Score for Each ADHD Symptom at Ages 7, 15 and 25 years separately for males and females

	Males			Females		
	Age 7	Age 15	Age 25	Age 7	Age 15	Age 25
Hyperactive-impulsive symptoms						
1: Fidgets	6.1 (4.7-7.4)	6.3 (3.8-8.7)	8.2 (5.9-10.6)	4.8 (3.0-6.6)	8.3 (4.7-11.8)	8.6 (4.4-12.7)
2: Difficulty staying seated	5.2 (3.7-6.6)	7.1 (4.0-10.3)	8.2 (5.2-11.2)	5.5 (3.3-7.7)	8.0 (3.3-12.7)	10.1 (8.1-12.2)
3: Runs about/climbs/ restless	6.9* (4.7-9.2)	-	5.9 (3.1-8.8)	6.0 (3.7-8.3)	-	5.3 (2.4-8.3)
4: Difficulty being quiet	6.0 (3.9-8.1)	7.7* (0.2-15.2)	8.9 (6.2-11.5)	5.8 (3.5-8.1)	10.3 (6.2-14.5)	10.2 (6.3-14.0)
5: Difficulty calming down/relaxing	6.4 (4.4-8.5)	7.8* (3.5-12.1)	7.3 (5.2-9.4)	4.7 (1.8-7.6)	11.6* (9.0-14.3)	8.5 (6.1-11.0)
6: Blurts out answers	4.4 (2.5-6.4)	6.0 (3.4-8.6)	9.8 (6.3-13.4)	6.4 (3.9-8.9)	10.8* (4.8-16.8)	12.2* (10.0-14.4)
7: Difficulty waiting their turn	7.6* (6.1-9.0)	7.0 (2.7-11.3)	10.6* (4.8-16.4)	7.2* (4.8-9.6)	-	10.9* (6.7-15.1)
8: Interrupts	5.6 (4.0-7.2)	6.1 (3.8-8.4)	8.6 (5.1-12.1)	4.6 (2.8-6.5)	9.1 (5.6-12.6)	11.3* (8.6-13.9)
9: Talks excessively	5.5 (4.1-6.9)	6.4 (4.2-8.5)	8.2 (4.9-11.5)	4.0 (2.5-5.6)	7.7 (4.2-11.1)	8.9 (3.8-13.9)
Inattentive symptoms						
10: Makes careless mistakes	6.4 (5.2-7.6)	7.6 (6.3-8.9)	9.5 (7.5-11.4)	6.0 (4.6-7.5)	9.9 (7.9-12.0)	9.3 (5.2-13.4)
11: Difficulty sustaining attention	7.3* (5.9-8.7)	8.6* (7.0-10.2)	11.8* (10.1-13.5)	6.4 (4.9-7.9)	10.5* (7.3-13.6)	9.1 (5.4-12.8)
12: Does not listen	6.6 (5.4-7.8)	6.1 (4.4-7.9)	11.4* (8.9-13.9)	5.3 (3.2-7.4)	9.5 (7.1-11.9)	8.1 (4.3-11.9)
13: Does not finish a task/job properly	6.8 (5.5-8.2)	7.7 (6.1-9.2)	10.4 (8.7-12.1)	6.9* (5.4-8.4)	9.6 (6.7-12.5)	7.5 (3.8-11.1)
14: Difficulty organising	6.3 (5.1-7.5)	7.5 (6.1-8.8)	9.0 (7.6-10.4)	4.2 (2.0-6.4)	7.7 (5.0-10.4)	8.8 (7.1-10.5)
15: Avoids concentration tasks	5.7 (4.5-6.9)	6.9 (5.3-8.6)	9.1 (6.6-11.8)	5.0 (3.8-6.3)	9.4 (6.4-12.3)	9.5 (7.6-11.4)
16: Loses things needed for school/work	5.6 (4.1-7.2)	6.4 (4.7-8.0)	9.3 (7.4-11.3)	7.2* (5.2-9.1)	9.2 (5.7-12.6)	9.1 (7.4-10.8)
17: Easily distracted	5.6 (4.5-6.7)	6.4 (4.7-8.0)	9.4 (7.3-11.4)	4.7 (3.1-6.2)	9.6 (7.2-12.0)	9.5 (8.1-10.8)
18: Forgetful	5.8 (4.5-7.2)	6.7 (5.2-8.3)	8.5 (6.3-10.7)	4.9 (2.5-7.2)	9.2 (6.4-12.0)	8.1 (6.5-9.6)

Note. Means derived using inverse probability weighting. 95% confidence intervals in parentheses. Items across age 7, 15 and 25 are the same but wording changes slightly at age 25 to be more appropriate to young adulthood. The asterisk (*) denotes the three most common symptoms at each age group. Means not reported where N<5.

Supplementary Table 6a.

Prevalence of Specific ADHD Symptoms at Ages 7, 15 and 25 years using different approaches to missing data: Hyperactive-impulsive symptoms

	Original estimate (IPW) N=2327			Complete cases N=2327			Any ADHD symptom data Total included N=8805		
	Age 7	Age 15	Age 25	Age 7	Age 15	Age 25	Age 7	Age 15	Age 25
Hyperactive-impulsive symptoms									
1: Fidgets	5.2%* (4.0-6.6)	3.0% (2.2-4.0)	1.7% (1.1-2.5)	4.3%* (3.5-5.2)	2.8% (2.2-3.5)	1.4% (1.0-1.9)	5.1%* (4.7-5.6)	3.3% (2.8-3.9)	1.5% (1.2-1.9)
2: Difficulty staying seated	4.8% (3.7-6.3)	2.1% (1.4-3.0)	1.4% (0.9-2.3)	3.7% (3.0-4.5)	1.8% (1.3-3.4)	1.1% (0.8-1.6)	4.8% (4.4-5.3)	2.3% (1.9-2.8)	1.4% (1.1-1.8)
3: Runs about/climbs/ restless	2.6% (1.7-3.7)	0.5% (0.2-1.4)	2.2% (1.5-3.3)	2.1% (1.6-2.8)	0.3% (0.1-0.6)	1.8% (1.3-2.4)	3.7% (3.3-4.1)	0.6% (0.4-0.9)	1.8% (1.5-2.3)
4: Difficulty being quiet	3.2% (2.3-4.4)	1.0% (0.6-1.9)	1.5% (0.9-2.4)	2.5% (1.9-3.2)	0.8% (0.5-1.2)	1.1% (0.8-1.6)	3.4% (3.1-3.9)	1.0% (0.8-1.4)	1.4% (1.1-1.9)
5: Difficulty calming down/relaxing	3.2% (2.3-4.5)	1.2% (0.7-2.1)	3.4%* (2.6-4.6)	2.4% (1.9-3.1)	0.7% (0.5-1.2)	2.9%* (2.3-3.7)	3.9% (3.5-4.3)	1.1% (0.8-1.4)	2.9%* (2.4-3.4)
6: Blurts out answers	2.4% (1.56-3.51)	2.2% (1.4-3.3)	0.5% (0.3-1.1)	1.6% (1.2-2.2)	1.5% (1.0-2.0)	0.5% (0.3-0.9)	2.4% (2.1-2.8)	1.9% (1.5-2.3)	0.7% (0.5-1.0)
7: Difficulty waiting their turn	2.35% (1.7-3.3)	1.4% (0.8-2.4)	0.7% (0.3-1.3)	2.1% (1.6-2.7)	1.0% (0.7-1.5)	0.6% (0.3-1.0)	3.3% (2.9-3.7)	1.4% (1.1-1.8)	0.8% (0.6-1.1)
8: Interrupts	4.3% (3.2-5.8)	2.7% (2.0-4.1)	1.0% (0.6-1.7)	3.2% (2.5-4.0)	1.8% (1.4-2.5)	0.8% (0.5-1.2)	4.6% (4.2-5.1)	2.4% (1.9-2.8)	1.1% (0.8-1.4)
9: Talks excessively	5.6%* (4.4-7.1)	3.4% (2.4-4.7)	1.3% (0.8-2.1)	4.3%* (3.5-5.2)	2.6% (2.0-3.3)	1.1% (0.7-1.6)	5.9%* (5.4-6.4)	3.2% (2.8-3.8)	1.4% (1.0-1.8)

Note. IPW = inverse probability weighting. The asterisk (*) denotes the three most common symptoms at each age group.

Supplementary Table 6b.

Prevalence of Specific ADHD Symptoms at Ages 7, 15 and 25 years using different approaches to missing data: Inattentive symptoms

	Original estimate (IPW) N=2327			Complete cases N=2327			Any ADHD symptom data Total included N=8805		
	Age 7	Age 15	Age 25	Age 7	Age 15	Age 25	Age 7	Age 15	Age 25
Inattentive symptoms									
10: Makes careless mistakes	3.6%	3.4%	0.9%	3.4%	3.3%	1.1%	4.2%	3.4%	1.2%
	(2.7-4.8)	(2.6-4.5)	(0.6-1.4)	(2.7-4.2)	(2.7-4.1)	(0.7-1.6)	(3.8-4.7)	(2.9-4.0)	(0.9-1.6)
11: Difficulty sustaining attention	3.1%	2.4%	0.9%	2.5%	2.0%	1.1%	3.4%	2.2%	1.2%
	(2.3-4.3)	(1.7-3.4)	(0.6-1.4)	(1.9-3.2)	(1.5-2.7)	(0.7-1.6)	(3.1-3.9)	(1.8-2.7)	(1.0-1.6)
12: Does not listen	5.0%	3.9%	1.3%	3.8%	2.8%	1.2%	4.8%	3.0%	1.4%
	(3.8-5.7)	(2.9-5.3)	(0.8-1.9)	(3.0-4.6)	(2.2-3.5)	(0.8-1.7)	(4.3-5.3)	(2.5-3.5)	(1.1-1.9)
13: Does not finish a task/job properly	2.7%	3.0%	1.4%	2.6%	2.5%	1.6%	3.5%	3.0%	1.5%
	(2.1-3.8)	(2.2-4.1)	(1.0-2.1)	(2.0-3.4)	(1.9-3.2)	(1.1-2.1)	(3.2-4.0)	(2.5-3.5)	(1.2-1.9)
14: Difficulty organising	3.6%	4.3%*	2.5%*	2.8%	4.2%*	2.5%*	3.9%	5.1%*	2.9%*
	(2.6-5.1)	(3.3-5.4)	(1.9-3.4)	(2.2-3.5)	(3.4-5.1)	(2.0-3.3)	(3.5-4.3)	(4.5-5.8)	(2.4-3.4)
15: Avoids concentration tasks	4.4%	4.1%*	1.9%	3.4%	3.4%*	1.8%	4.4%	4.4%*	2.0%
	(3.4-5.7)	(3.1-5.3)	(1.3-2.7)	(2.7-4.)	(2.8-4.3)	(1.3-2.4)	(4.0-4.9)	(3.8-5.0)	(1.6-2.4)
16: Loses things needed for school/work	1.9%	2.9%	2.2%	1.6%	3.1%	2.0%	2.5%	3.9%	2.0%
	(1.3-2.8)	(2.2-3.9)	(1.6-3.1)	(1.2-2.2)	(2.5-3.9)	(1.5-2.7)	(2.2-2.8)	(3.4-4.5)	(1.6-2.4)
17: Easily distracted	6.0%*	4.4%*	2.5%*	4.5%*	3.4%*	2.2%*	5.8%*	4.4%*	2.3%*
	(4.7-7.7)	(3.4-5.8)	(1.8-3.4)	(3.7-5.4)	(2.8-4.3)	(1.7-2.9)	(5.3-6.3)	(3.8-5.1)	(1.8-2.8)
18: Forgetful	3.3%	3.8%	2.1%	2.5%	3.4%	2.0%	3.5%	3.9%	2.2%
	(2.4-4.4)	(2.9-5.0)	(1.5-2.9)	(2.0-3.3)	(2.7-4.2)	(1.5-2.6)	(3.1-3.9)	(3.3-4.5)	(1.8-2.7)

Note. IPW = inverse probability weighting. The asterisk (*) denotes the three most common symptoms at each age group.

Supplementary Table 7a.

Mean Impairment Score for Each ADHD Symptom at Ages 7, 15 and 25 years using different approaches to missing data: Hyperactive-impulsive symptoms

	Original estimate (IPW)			Complete cases			Any ADHD symptom data		
	Age 7	Age 15	Age 25	Age 7	Age 15	Age 25	Age 7	Age 15	Age 25
Hyperactive-impulsive symptoms									
1: Fidgets	5.6 (4.5-6.7)	6.9 (4.9-8.9)	8.3 (6.4-10.3)	5.8 (5.0-6.7)	6.4 (4.9-7.8)	7.9 (5.9-9.8)	6.6 (6.2-7.1)	7.3 (6.3-8.2)	8.5 (7.0-9.9)
2: Difficulty staying seated	5.3 (4.1-6.4)	7.5 (5.0-9.9)	9.0 (7.2-10.8)	5.7 (4.8-6.6)	7.1 (5.3-9.0)	8.6 (6.8-10.7)	6.7 (6.2-7.2)	7.9 (6.7-9.0)	9.1 (7.8-10.5)
3: Runs about/climbs/ restless	6.5 (4.9-8.1)	7.7 (-0.0-16.3)	5.7 (3.7-7.6)	6.1 (4.8-7.3)	8.2 (1.5-14.9)	5.1 (3.2-6.9)	7.1 (6.6-7.6)	10.0* (7.9-12.2)	5.9 (4.5-7.3)
4: Difficulty being quiet	5.9 (4.4-7.5)	8.8* (5.0-12.5)	9.3 (7.2-11.4)	6.2 (5.2-7.2)	9.0* (6.1-11.9)	8.8 (6.7-10.9)	7.5 (7.0-8.0)	9.5* (7.9-11.0)	9.7 (8.4-11.0)
5: Difficulty calming down/relaxing	5.8 (4.1-7.5)	8.6* (6.0-11.9)	7.8 (6.2-9.3)	6.2 (5.0-7.4)	9.3* (7.1-11.5)	7.3 (6.0-8.6)	7.2 (6.7-7.6)	9.3* (7.8-10.9)	8.2 (7.2-9.2)
6: Blurts out answers	5.0 (3.4-6.7)	6.7 (4.2-9.1)	11.1* (8.9-13.2)	5.9 (4.7-7.2)	7.6 (5.8-9.5)	10.3 (8.2-12.3)	7.2 (6.6-7.8)	8.5 (7.4-9.6)	11.3* (10.1-12.5)
7: Difficulty waiting their turn	7.5* (6.3-8.7)	7.4 (3.6-11.1)	10.8* (7.9-13.7)	6.8* (5.7-8.9)	7.8 (5.4-10.3)	11.0* (8.3-13.7)	7.5* (7.0-8.1)	9.1 (7.8-10.4)	11.3* (9.8-12.8)
8: Interrupts	5.2 (4.0-6.4)	6.7 (4.7-8.7)	9.8 (7.4-12.1)	5.8 (4.9-6.6)	7.3 (5.8-8.8)	9.9 (7.7-12.2)	6.7 (6.3-7.2)	7.9 (6.9-9.0)	10.8* (9.6-12.0)
9: Talks excessively	5.0 (3.9-6.0)	6.8 (5.1-8.5)	8.6 (5.8-11.3)	5.5 (4.7-6.3)	7.5 (6.2-8.9)	8.2 (6.1-10.3)	6.2 (5.8-6.3)	7.9 (7.0-8.7)	9.9 (8.6-11.2)

Note. IPW = inverse probability weighting. The asterisk (*) denotes the three most common symptoms at each age group.

Supplementary Table 7b.

Mean Impairment Score for Each ADHD Symptom at Ages 7, 15 and 25 years using different approaches to missing data: Inattentive symptoms

	Original estimate (IPW)			Complete cases			Any ADHD symptom data		
	Age 7	Age 15	Age 25	Age 7	Age 15	Age 25	Age 7	Age 15	Age 25
Inattentive symptoms									
10: Makes careless mistakes	6.3	8.3	9.4	6.2	7.7	10.0	7.4	8.1	10.4
	(5.4-7.2)	(7.1-9.4)	(7.4-11.3)	(5.4-7.0)	(6.7-8.7)	(8.6-11.4)	(6.6-7.5)	(7.4-8.8)	(9.3-11.4)
11: Difficulty sustaining attention	7.0*	9.1*	10.5*	7.1*	8.6*	10.8*	7.6*	9.2	10.8
	(6.0-8.0)	(7.7-10.4)	(8.4-12.7)	(6.2-8.0)	(7.3-9.9)	(9.5-12.1)	(7.2-8.1)	(8.3-10.1)	(9.8-11.8)
12: Does not listen	6.1	6.9	9.6	6.5	7.5	10.7*	7.0	8.1	10.2
	(5.0-7.2)	(5.3-8.5)	(7.1-12.1)	(5.7-7.2)	(6.4-8.6)	(9.3-12.2)	(6.6-7.4)	(7.3-8.9)	(9.0-11.4)
13: Does not finish a task/job properly	6.9*	8.2	9.1	6.9*	7.7	9.6	7.6*	7.9	10.2
	(5.9-7.9)	(6.8-9.6)	(6.9-11.1)	(6.1-7.8)	(6.5-8.9)	(8.2-11.1)	(7.1-8.0)	(7.2-8.7)	(9.0-11.3)
14: Difficulty organising	5.5	7.5	8.9	6.2	7.3	8.9	7.2	7.2	9.3
	(4.3-6.8)	(6.3-8.7)	(7.9-10.0)	(5.3-7.0)	(6.4-8.2)	(7.9-10.0)	(6.8-7.6)	(6.6-7.8)	(8.6-10.0)
15: Avoids concentration tasks	5.4	7.5	9.3	5.7	7.9	10.1	6.4	7.6	9.9
	(4.6-6.3)	(6.0-9.0)	(7.8-10.9)	(5.0-6.4)	(6.9-8.8)	(9.1-11.1)	(6.0-6.8)	(6.9-8.2)	(9.1-10.8)
16: Loses things needed for school/work	6.2	6.9	9.2	6.4	7.0	9.2	6.9	7.2	9.4
	(4.9-7.4)	(5.3-8.5)	(8.0-10.5)	(5.3-7.4)	(5.9-8.2)	(8.1-10.3)	(6.3-7.4)	(6.5-7.9)	(8.5-10.3)
17: Easily distracted	5.2	7.1	9.4	5.8	7.7	9.8	6.5	7.7	9.8
	(4.3-6.2)	(5.6-8.5)	(8.3-10.6)	(5.1-6.4)	(6.7-8.6)	(8.9-10.8)	(6.1-6.8)	(7.1-8.3)	(9.0-10.6)
18: Forgetful	5.5	7.12	8.3	6.2	7.2	8.3	6.9	7.2	8.9
	(4.3-6.7)	(5.7-8.6)	(7.0-9.5)	(5.3-7.1)	(6.2-8.3)	(7.2-9.5)	(6.4-7.4)	(6.5-7.9)	(8.1-9.8)

Note. IPW = inverse probability weighting. The asterisk (*) denotes the three most common symptoms at each age group.

Supplementary Table 8.

Tests of measurement invariance across age

Model	Free parameters	CFI	RMSEA (90% CI)	SRMR	vs.	Δ parameters	ΔCFI	ΔRMSEA	ΔSRMR	Decision
A: Configural invariance	123	0.995	0.007 (0.007-0.008)	0.055	-					
B: Metric invariance	91	0.994	0.008 (0.007-0.009)	0.063	A	32	0.001	-0.001	-0.008	Accept
C: Scalar invariance	91	0.994	0.008 (0.007-0.009)	0.055	-					
D: Residual invariance	55	0.992	0.009 (0.008-0.010)	0.062	B	36	0.002	-0.001	0.001	Accept
D: Residual invariance	55	0.992	0.009 (0.008-0.010)	0.062	C	36	0.002	-0.001	-0.007	Accept

Note. Residual variances fixed to 1 for models A and B. CFI = comparative fit index, RMSEA = root-mean-square error of approximation, SRMR = standardized root mean squared residual.

Supplementary Table 9.

Associations between missing ADHD data and variables included in the inverse probability weight (IPW) multiple imputation model

	Initial univariable analyses	Final multivariable analysis for IPW*
Male sex	OR=1.32, 95% CI=1.21-1.45	OR=1.35, 95% CI=1.23-1.49
Home ownership	OR=0.30, 95% CI=0.26-0.34	OR=0.53, 95% CI=0.46-0.61
Maternal depression	OR=1.97, 95% CI=1.62-2.40	OR=1.44, 95% CI=1.18-1.77
Maternal age at birth	OR=0.91, 95% CI=0.90-0.92	OR=0.93, 95% CI=0.92-0.94
Maternal education	OR=0.62, 95% CI=0.59-0.64	OR=0.68, 95% CI=0.65-0.71
Parity	OR=1.21, 95% CI=1.15-1.27	OR=1.18, 95% CI=1.11-1.24

Note. Missing data on indicators used in final IPW model were singly imputed as the modal or mean value (all <15% missing).

References

American Psychiatric Association. (2013). Attention-Deficit/Hyperactivity Disorder (ADHD). In *Diagnostic and Statistical Manual of Mental Disorders* (5th ed.).

Barkley, R. A. (2011). *Barkley Adult ADHD Rating Scale-IV (BAARS-IV)*. Guilford Press.

Cheung, G. W., & Rensvold, R. B. (2002). Evaluating Goodness-of-Fit Indexes for Testing Measurement Invariance. *Structural Equation Modeling: A Multidisciplinary Journal*, *9*(2), 233-255. https://doi.org/10.1207/S15328007SEM0902_5

Goodman, A., Heiervang, E., Collishaw, S., & Goodman, R. (2011). The 'DAWBA bands' as an ordered-categorical measure of child mental health: description and validation in British and Norwegian samples. *Social psychiatry and psychiatric epidemiology*, *46*(6), 521-532.

Hu, L. T., & Bentler, P. M. (1999). Cutoff Criteria for Fit Indexes in Covariance Structure Analysis: Conventional Criteria Versus New Alternatives. *Structural Equation Modeling-a Multidisciplinary Journal*, *6*(1), 1-55. https://doi.org/10.1080/10705519909540118

Muthén, L. K., & Muthén, B. O. (1998-2012). *Mplus User's Guide* (Seventh ed.). Muthén & Muthén.

Putnick, D. L., & Bornstein, M. H. (2016). Measurement invariance conventions and reporting: The state of the art and future directions for psychological research. *Developmental review*, *41*, 71-90.

Sayon-Orea, C., Moreno-Iribas, C., Delfrade, J., Sanchez-Echenique, M., Amiano, P., Ardanaz, E., Gorricho, J., Basterra, G., Nuin, M., & Guevara, M. (2020). Inverse-probability weighting and multiple imputation for evaluating selection bias in the estimation of childhood obesity prevalence using data from electronic health records. *BMC Medical Informatics and Decision Making*, *20*(1), 9. https://doi.org/10.1186/s12911-020-1020-8

Seaman, S. R., & White, I. R. (2013). Review of inverse probability weighting for dealing with missing data. *Stat Methods Med Res*, *22*(3), 278-295. https://doi.org/10.1177/0962280210395740

Seaman, S. R., White, I. R., Copas, A. J., & Li, L. (2012). Combining multiple imputation and inverse-probability weighting. *Biometrics*, *68*(1), 129-137. https://doi.org/10.1111/j.1541-0420.2011.01666.x
